# Supplementary material for: Modeling glioblastoma heterogeneity as a dynamic network of cell states
Source: Mol Syst Biol. 2021 Sep 16;17(9):e10105. doi: 10.15252/msb.202010105 (PMC8444284; doi:10.15252/msb.202010105)
Supplement: Supplementary file 6 — Source Data for Figure 5 [file MSB-17-e10105-s004.zip › Figure5A_sourcedata/GSEA_3071/hallmarks_stateVI.GseaPreranked.1623742557031/gsea_report_for_na_neg_1623742557031.html]

Report for na\_neg 1623742557031 [GSEA]

| GS  follow link to MSigDB | GS DETAILS | SIZE | ES | NES | NOM p-val | FDR q-val | FWER p-val | RANK AT MAX | LEADING EDGE || 1 | HALLMARK\_MYOGENESIS | Details ... | 57 | -0.43 | -1.96 | 0.001 | 0.043 | 0.068 | 603 | tags=35%, list=17%, signal=41% |
| 2 | HALLMARK\_KRAS\_SIGNALING\_DN | Details ... | 25 | -0.53 | -1.95 | 0.004 | 0.023 | 0.075 | 982 | tags=64%, list=27%, signal=87% |
| 3 | HALLMARK\_E2F\_TARGETS | Details ... | 98 | -0.34 | -1.70 | 0.009 | 0.130 | 0.479 | 1049 | tags=51%, list=29%, signal=70% |
| 4 | HALLMARK\_ANGIOGENESIS | Details ... | 15 | -0.51 | -1.66 | 0.033 | 0.139 | 0.605 | 515 | tags=40%, list=14%, signal=46% |
| 5 | HALLMARK\_ESTROGEN\_RESPONSE\_LATE | Details ... | 61 | -0.35 | -1.60 | 0.043 | 0.155 | 0.734 | 765 | tags=41%, list=21%, signal=51% |
| 6 | HALLMARK\_KRAS\_SIGNALING\_UP | Details ... | 54 | -0.35 | -1.59 | 0.032 | 0.145 | 0.778 | 360 | tags=28%, list=10%, signal=30% |
| 7 | HALLMARK\_HEDGEHOG\_SIGNALING | Details ... | 17 | -0.46 | -1.55 | 0.064 | 0.155 | 0.832 | 968 | tags=53%, list=27%, signal=72% |
| 8 | HALLMARK\_G2M\_CHECKPOINT | Details ... | 90 | -0.29 | -1.40 | 0.070 | 0.302 | 0.985 | 864 | tags=41%, list=24%, signal=53% |
| 9 | HALLMARK\_SPERMATOGENESIS | Details ... | 23 | -0.36 | -1.32 | 0.180 | 0.403 | 0.998 | 1455 | tags=74%, list=40%, signal=122% |
| 10 | HALLMARK\_APOPTOSIS | Details ... | 70 | -0.27 | -1.29 | 0.182 | 0.405 | 0.998 | 941 | tags=41%, list=26%, signal=55% |
| 11 | HALLMARK\_BILE\_ACID\_METABOLISM | Details ... | 17 | -0.38 | -1.24 | 0.240 | 0.459 | 1.000 | 780 | tags=41%, list=21%, signal=52% |
| 12 | HALLMARK\_COMPLEMENT | Details ... | 46 | -0.27 | -1.16 | 0.296 | 0.560 | 1.000 | 278 | tags=20%, list=8%, signal=21% |
| 13 | HALLMARK\_COAGULATION | Details ... | 36 | -0.28 | -1.13 | 0.318 | 0.559 | 1.000 | 410 | tags=25%, list=11%, signal=28% |
| 14 | HALLMARK\_MITOTIC\_SPINDLE | Details ... | 70 | -0.24 | -1.12 | 0.296 | 0.543 | 1.000 | 1225 | tags=46%, list=34%, signal=68% |
| 15 | HALLMARK\_HEME\_METABOLISM | Details ... | 46 | -0.23 | -1.03 | 0.423 | 0.673 | 1.000 | 1188 | tags=46%, list=33%, signal=67% |
| 16 | HALLMARK\_P53\_PATHWAY | Details ... | 75 | -0.20 | -0.96 | 0.533 | 0.752 | 1.000 | 587 | tags=23%, list=16%, signal=26% |
| 17 | HALLMARK\_CHOLESTEROL\_HOMEOSTASIS | Details ... | 21 | -0.27 | -0.95 | 0.514 | 0.718 | 1.000 | 585 | tags=29%, list=16%, signal=34% |
| 18 | HALLMARK\_PI3K\_AKT\_MTOR\_SIGNALING | Details ... | 36 | -0.24 | -0.94 | 0.524 | 0.690 | 1.000 | 338 | tags=17%, list=9%, signal=18% |
| 19 | HALLMARK\_PEROXISOME | Details ... | 20 | -0.26 | -0.89 | 0.572 | 0.733 | 1.000 | 1171 | tags=45%, list=32%, signal=66% |
| 20 | HALLMARK\_IL6\_JAK\_STAT3\_SIGNALING | Details ... | 18 | -0.25 | -0.84 | 0.637 | 0.768 | 1.000 | 515 | tags=22%, list=14%, signal=26% |
| 21 | HALLMARK\_UV\_RESPONSE\_UP |  | 47 | -0.20 | -0.84 | 0.676 | 0.737 | 1.000 | 1313 | tags=45%, list=36%, signal=69% |
| 22 | HALLMARK\_DNA\_REPAIR |  | 51 | -0.15 | -0.66 | 0.870 | 0.909 | 1.000 | 1075 | tags=35%, list=30%, signal=49% |
| 23 | HALLMARK\_PROTEIN\_SECRETION |  | 25 | -0.14 | -0.52 | 0.951 | 0.962 | 1.000 | 55 | tags=4%, list=2%, signal=4% |
Table: Gene sets enriched in phenotype **na **[plain text format]****
